# Thoracoscopic Direct Suture Closure for a Large Bulla With Severe Adhesion Under Local Anesthesia: A Case Report

**DOI:** 10.7759/cureus.54718

**Published:** 2024-02-22

**Authors:** Naoya Kitamura, Tomohiko Takahashi, Jun Kawamukai, Hideki Shinno, Tomoshi Tsuchiya

**Affiliations:** 1 Thoracic Surgery, Toyama University Hospital, Toyama, JPN; 2 General Thoracic Surgery, Toyama Prefectural Central Hospital, Toyama, JPN

**Keywords:** emphysema, thoracoscopic surgery, secondary spontaneous pneumothorax, local anesthesia, bulla suture

## Abstract

Although the usefulness of thoracoscopic surgery under local anesthesia for pneumothorax has been reported, there are some cases of failure. Therefore, it is important to share the various techniques and potential challenges associated with procedures performed under local anesthesia. A 79-year-old male, under monitoring for a left chronic pneumothorax, was newly diagnosed with a right pneumothorax. Chest computed tomography taken after thoracic drainage showed a poorly expanded right lung with severe adhesions and multiple bullae in the right lung, in addition to identifying a left pneumothorax. Although significant air leakage persisted, general anesthesia was deemed unsuitable, necessitating thoracoscopic surgery under local anesthesia. A fistula of approximately 1 × 1 cm was identified on the bulla wall, which was closed with 4-0 Prolene®sutures (Johnson&Johnson, New Jersey, United States), each reinforced with pledgets and covered with a polyglycolic acid sheet and fibrin glue. The patient was discharged on postoperative day six and no recurrence of pneumothorax was noted after discharge. Direct suture closure of the bulla wall under local anesthesia can be an alternative technique for the treatment of pneumothorax caused by large bulla collapse in patients at high risk for general anesthesia.

## Introduction

Pneumothorax is a condition in which air leaks into the thoracic cavity due to damage to visceral pleura, with primary and secondary spontaneous pneumothorax being the most common causes. The former is usually caused by a ruptured bulla, while the latter may occur in high-risk patients, such as those with severe chronic obstructive pulmonary disease (COPD) or patients with impaired respiratory function. However, it may be difficult to perform the procedure under general anesthesia for such patients because of the maintenance of one-lung ventilation or the associated risk of contralateral pneumothorax [1-3]. Although several treatment options, such as pleurodesis [4] or insertion of an endobronchial Watanabe spigot (EWS) [5,6] have been reported, these methods are difficult to perform and sometimes unsuccessful [6]. In contrast, the usefulness of thoracoscopic surgery under local anesthesia for pneumothorax has been reported in many cases [7-9]. In addition, the reported surgical techniques are limited to relatively easy procedures such as fibrin glue coverage [10,11], talc pleurodesis [12-14], cyst ligation [15], and bulla resection using a stapler [16,17]. However, the limitations of these procedures have resulted in treatment failures in some cases [18].

In this report, we describe a surgical technique for direct suture closure of severely adherent bullae under local anesthesia in patients with bilateral pneumothorax developed from severe emphysema, and the factors that contribute to its success.

## Case presentation

**Figure 1:**
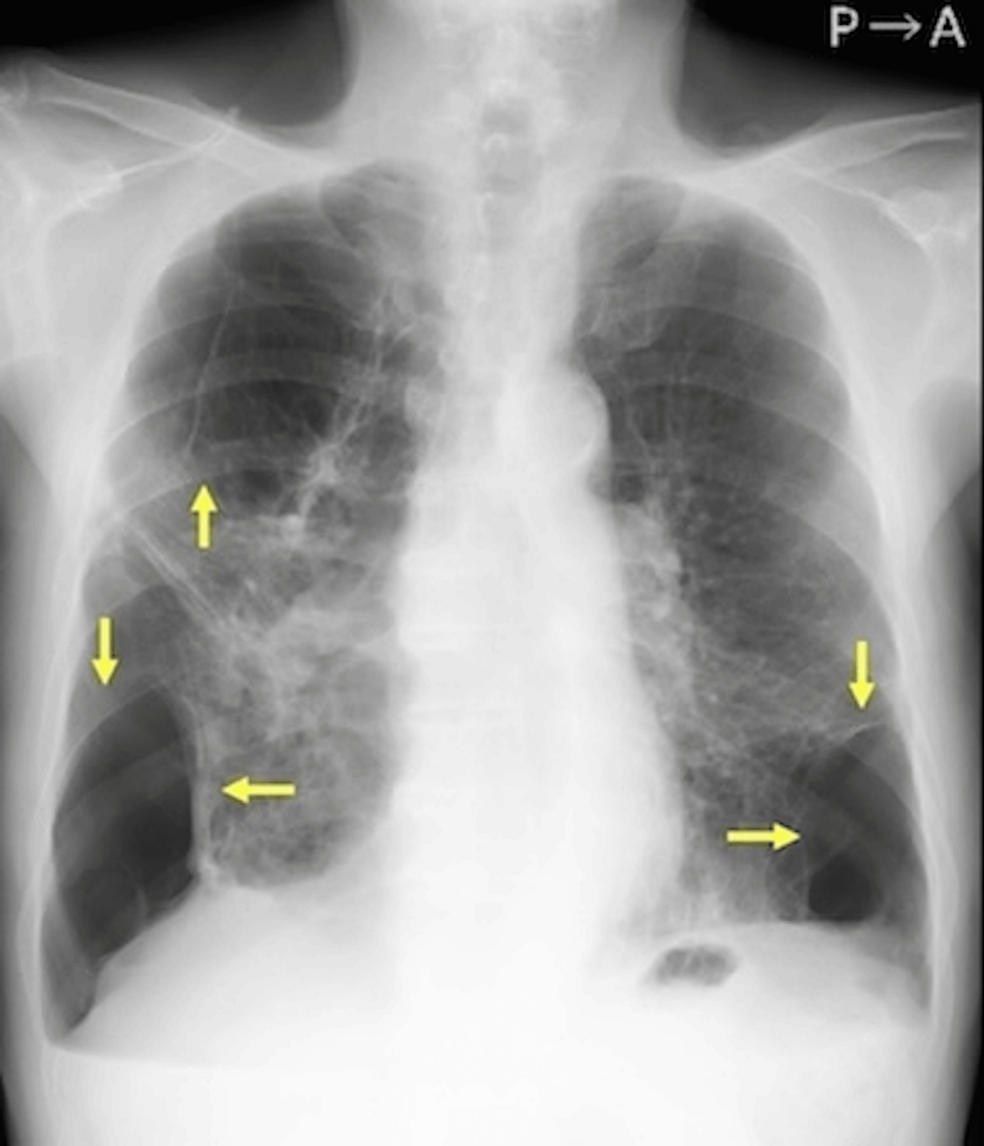
Chest radiograph Chest radiograph showed increased permeability in the right upper lung field and bilateral lower lung fields, suggesting a pneumothorax cavity (yellow arrows).

**Figure 2:**
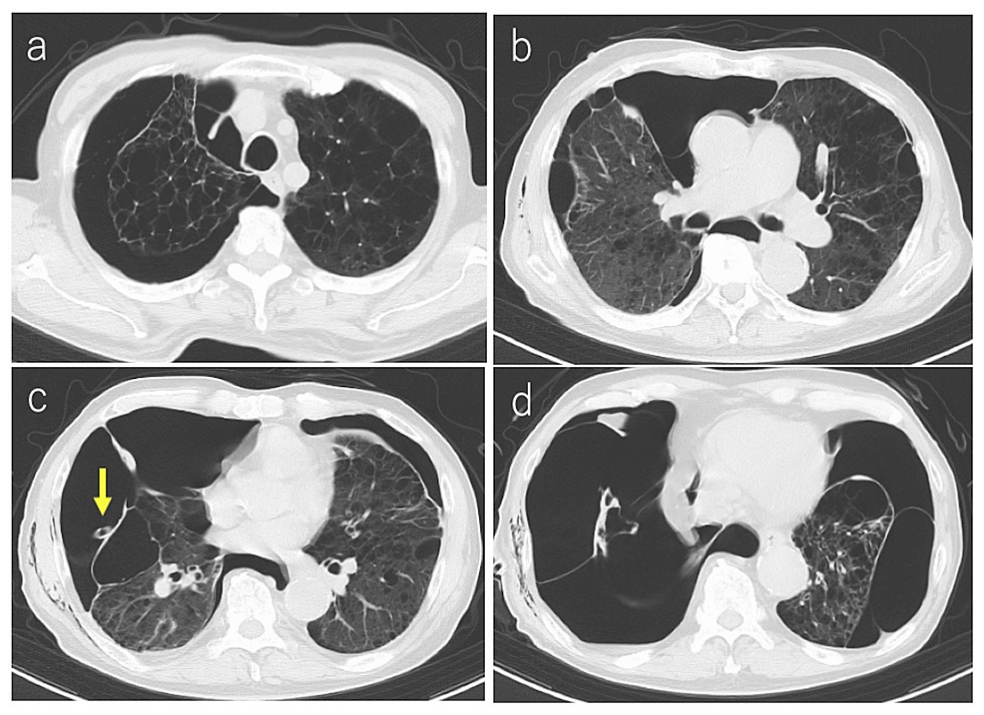
Chest computed tomography (a,b) Chest computed tomography showed marked emphysematous changes in bilateral lungs with severe adhesions. (c,d) A thoracic drain (yellow arrow) was placed, but the right lung was poorly dilated. A pneumothorax cavity was also present in the left lung, and the lower lobe of the left lung had emphysematous changes with a bulla. It was difficult to observe the fistula in the right lung.

A 79-year-old male who had a history of pneumothorax with pleurodeses, twice on the left side and once on the right side, was under observation for a left chronic pneumothorax. Additionally, a new diagnosis of right pneumothorax was confirmed through a chest radiograph (Figure 1). He was admitted to our hospital, and thoracic drainage was initiated. Chest computed tomography imaging showed marked emphysematous changes in bilateral lungs with severe adhesions and multiple bullae of various sizes (Figure 2a-2b). In addition, the expansion of the right lung was poor, and the left pneumothorax was sustained (Figure 2c-2d). Conservative therapy was deemed to be difficult because of poor expansion of the right lung as a result of a significant air leak. We initially considered thoracoscopic surgery under general anesthesia for pulmonary fistula closure or pleurodesis. However, we decided to perform thoracoscopic surgery under local anesthesia because of pulmonary dysfunction, which might make the patient unable to tolerate one-lung ventilation and worsening contralateral pneumothorax.

**Figure 3:**
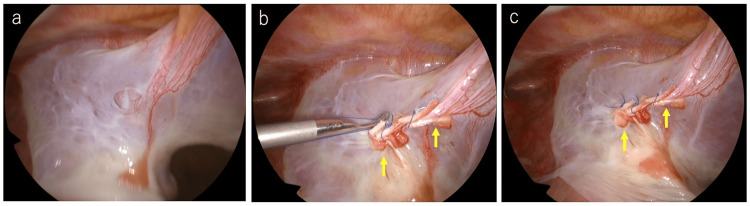
Intraoperative findings (a) Intraoperative findings revealed an approximately 1 × 1 cm hole in the bulla wall. The bulla itself was large with extensive adhesions. (b,c) The air leak point was closed with three stitches using 4-0 Prolene® sutures (Johnson & Johnson, New Jersey, United States), each reinforced with pledgets (yellow arrows).

**Figure 4:**
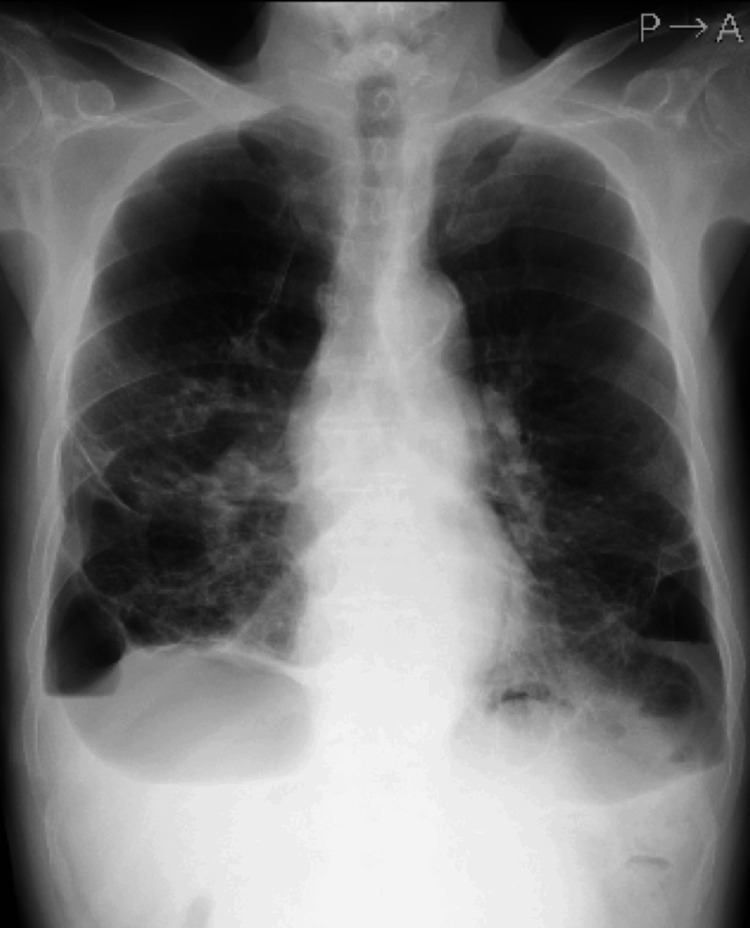
Chest radiograph on postoperative day 19 Chest radiograph showed increased permeability of the right lower lung field due to the bullae, but the right lung was well dilated.

Three-port thoracoscopic surgery was performed under local anesthesia. Extensive adhesions and multiple bullae were observed in the right thoracic cavity. An air leak test identified an approximately 1 × 1 cm fistula in the bulla (Figure 3a). Due to the considerable size of the bulla and its extensive adhesions, suture dissection with a stapler or ligation of the bulla base seemed difficult. Further, we considered that the fistula could not be closed by fibrin glue coverage or talc pleurodesis; hence, a direct suture of the fistula was indicated. The fistula of the air leak point was closed with three stitches using 4-0 Prolene® sutures (Johnson & Johnson, New Jersey, United States) on the bulla wall with pledgets and covered with a polyglycolic acid (PGA) sheet and fibrin glue (Figure 3b-3c). Lidocaine (2 mL) was used for local injection into the port wounds. The patient was able to maintain his position in an awake state and vital signs were stable with small doses of oxygen (Pulse: 75 bpm, Blood pressure: 120/75 mmHg, oxygen saturation (SpO2): 90% (nasal oxygen 1L). Pain and cough were easily managed with local anesthesia only, and no transvenous sedatives or analgesics were used. Air leakage ceased after surgery, and a chest radiograph showed good expansion of the right lung. Because a recurrent pneumothorax was a potentially serious situation for this patient, after careful follow-up, the thoracic drain was removed on postoperative day five, and the patient was discharged on postoperative day six. There was no recurrence of pneumothorax, and a chest radiograph on postoperative day 19 showed good lung expansion (Figure 4).

## Discussion

Although pneumothorax often occurs in patients with severe COPD, emphysema, or reduced respiratory function, general anesthesia can be difficult because one-lung ventilation sometimes fails to maintain oxygenation and requires two-lung ventilation [1] or causes contralateral pneumothorax [2,3]. In such high-risk cases, several treatment options have been reported. First, non-surgical methods such as pleurodesis [4] or insertion of an EWS [5,6] are considered; however, these methods are difficult to perform and sometimes unsuccessful [6]. Another option is thoracoscopic surgery under venovenous-extracorporeal membrane oxygenation (VV-ECMO) to ensure oxygenation [19], but this procedure is highly invasive [20]. Therefore, thoracoscopic surgery under local anesthesia, which is relatively safe and effective, was selected in this case.

The usefulness of thoracoscopic surgery under local anesthesia for high-risk pneumothorax has been reported in several cases [7-9]. Most of the reported techniques are relatively easy to perform, such as covering with fibrin glue and PGA sheets [10,11], talc pleurodesis [12-14], cyst ligation [15], and bulla resection using a stapler [16,17]. However, in some reported cases, air leaks couldn’t be stopped, and additional surgery under local anesthesia or general anesthesia was performed [11]. Depending on the localization and size of the bulla, as well as the degree of adhesion and location of the air leak point, the above procedures may be inapplicable, and it is necessary to pursue an alternative technique. In this case, the base of the bulla was wide, with extensive adhesions and little mobility. Furthermore, the fisture was relatively large, making closure by the above technique difficult. There are multiple reasons for the success of this surgical technique. First, it was easy to maintain rest, pain control, and cough reflex. Additional reasons include the ability to place the port in a straight line toward the leak point, good visual field, and the use of a prejet, which prevents re-rupture of the bulla wall. 

The direct suturing of the bulla wall under local anesthesia was suggested to be an effective technique contributing to the cessation of air leaks and early discharge from the hospital when the conditions are favorable as mentioned above. In addition, this technique can be considered a treatment option for those at high risk of pneumothorax due to large bulla failure.

## Conclusions

The direct suture closure of the bulla wall under local anesthesia is an alternative technique for the treatment of pneumothorax caused by large bulla collapse in patients at high risk for general anesthesia. However, favorable surgical conditions, such as maintenance of the patient's condition and good field of view of the thoracic cavity, are important for the success of this procedure. A prospective comparative study of a large number of patients is needed to further emphasize these results.
